# Supplementary material for: The P-type ATPase transporter ATP7A promotes angiogenesis by limiting autophagic degradation of VEGFR2
Source: Nat Commun. 2021 May 25;12:3091. doi: 10.1038/s41467-021-23408-1 (PMC8149886; doi:10.1038/s41467-021-23408-1)
Supplement: Supplementary file 1 — Supplementary Information [file 41467_2021_23408_MOESM1_ESM.pdf]

Supplementary information

The P-type ATPase transporter ATP7A promotes angiogenesis by limiting autophagic degradation of VEGFR2

Dipankar Ash, Varadarajan Sudhahar, Seock-Won Youn, Mustafa Nazir Okur, Archita Das,
John P. O'Bryan, Maggie McMenamin, Yali Hou, Jack H. Kaplan
Tohru Fukai, Masuko Ushio-Fukai

These authors contributed equally: Dipankar Ash, Varadarajan Sudhahar

These authors jointly supervised this work: Tohru Fukai and Masuko Ushio-Fukai

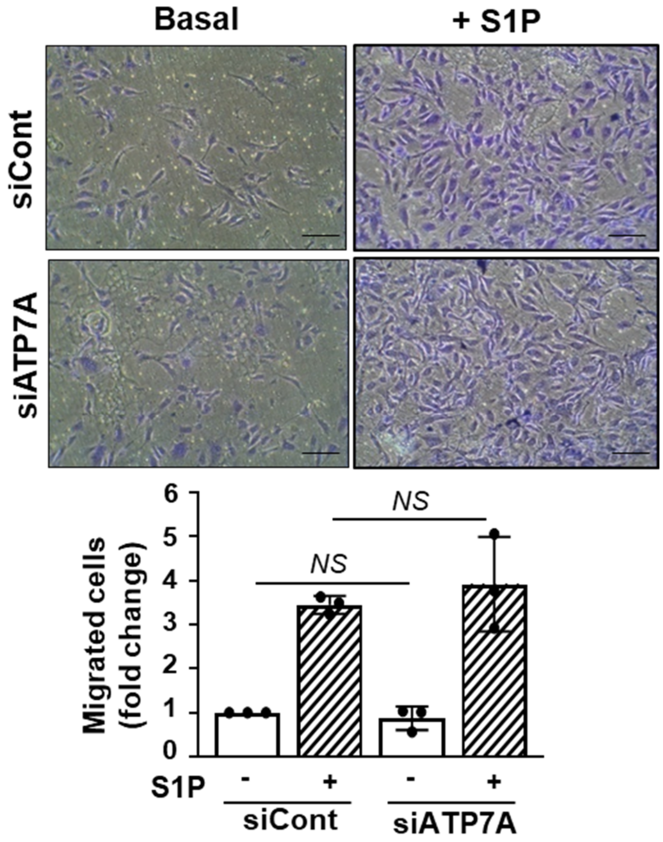

Supplementary Fig. 1: ATP7A has no effect on EC migration induced by sphingosine 1-phosphate (S1P): Human umbilical vein endothelial cells (HUVECs) transfected with control or ATP7A siRNAs were stimulated with S1P (10μM) for 6h. ECs migration was measured by modified Boyden chamber method. Bar graph represents averaged migrated cells, expressed as fold change over control. n=3, NS=non-significant (two-tailed unpaired t-test). Scale bars=100 μm. Data are mean ± SEM.

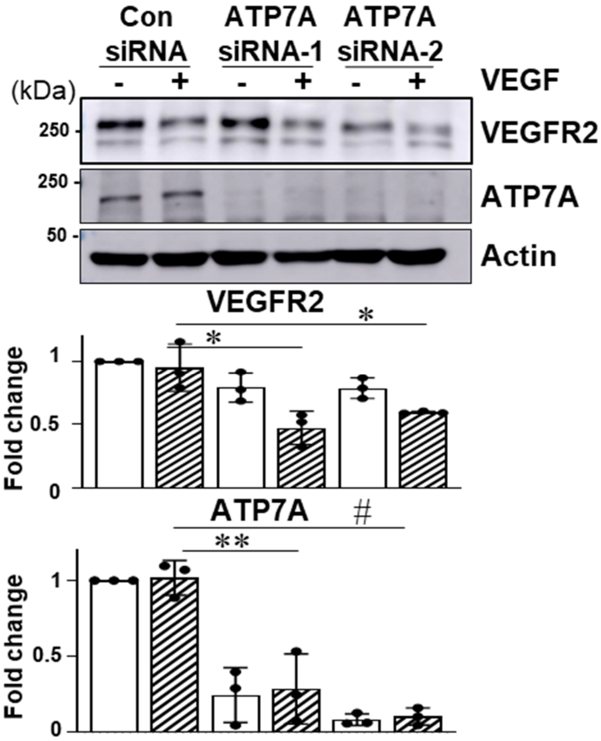

Supplementary Fig. 2: ATP7A knockdown promotes VEGF-induced VEGFR2 degradation in bovine aortic ECs: Bovine aortic endothelial cells (BAEC) were transfected with control or ATP7A siRNAs targeting different region of ATP7A mRNA, serum starved overnight followed by stimulation with VEGF (20ng/ml) for 30 min. Lysates were immunoblotted with indicated antibodies. β-actin is loading control. n=3, VEGFR2: *p=0.0229, *p=0.0309; ATP7A: **p=0.0019, #p<0.0001 (two-tailed unpaired t-test). Data are mean ± SEM.

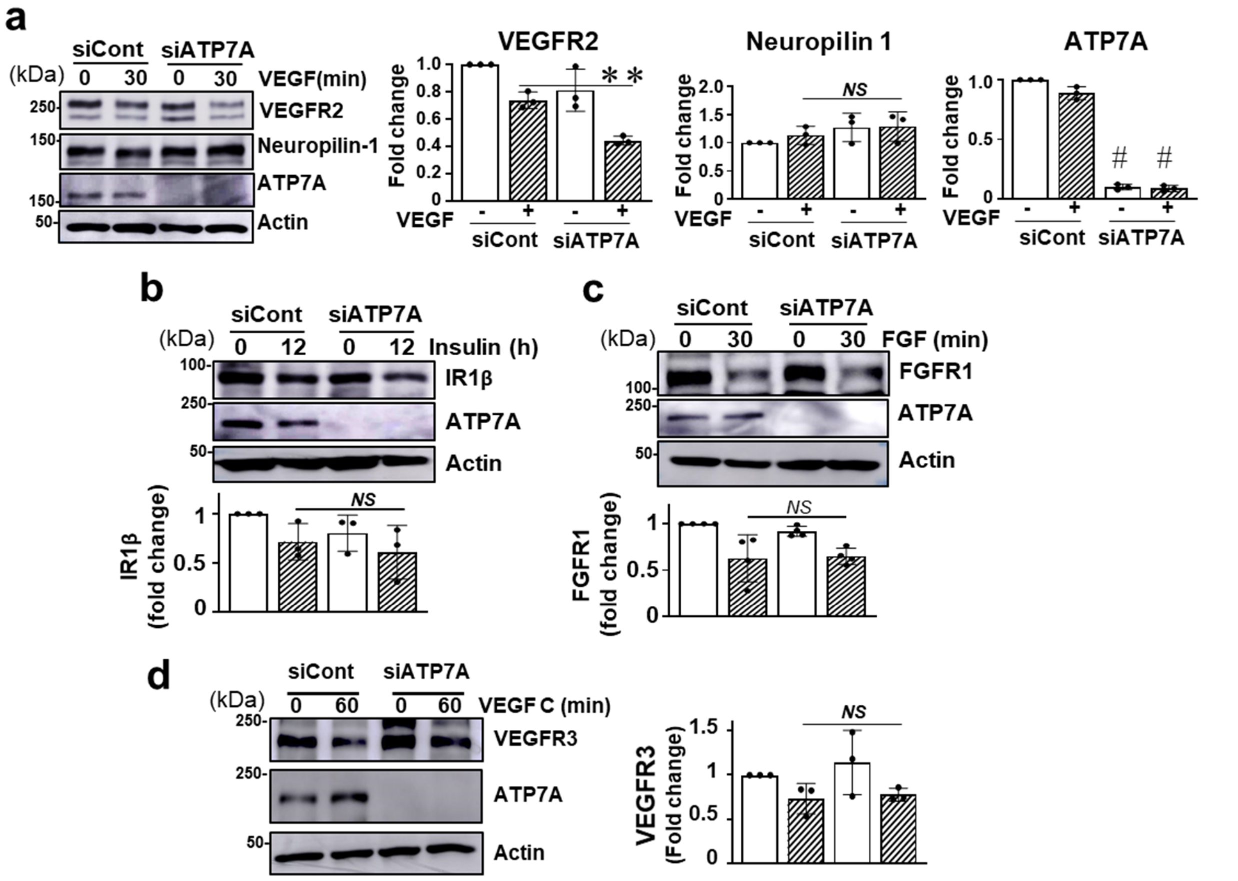

Supplementary Fig. 3: ATP7A knockdown has no effect on protein expression of Neuropilin, IR1β, FGFR1 and VEGFR3 in ECs: a-d: Human umbilical vein endothelial cells (HUVECs) transfected with control or ATP7A siRNAs were serum starved for 3h, followed by vascular endothelial growth factor (VEGF) stimulation for 30 min **(a)** or insulin (100nM) stimulation for 12h **(b)** or FGF (30ng/ml) stimulation for 30 min **(c)** or VEGF-C (100 ng/ml) for 1h **(d)**. Lysates were immunoblotted with indicated antibodies. In **a**, VEGFR2: **p=0.0017; Neuropilin: NS=non-significant; ATP7A: #p<0.0001 (two-tailed unpaired t-test). Data are mean ± SEM. N=3.

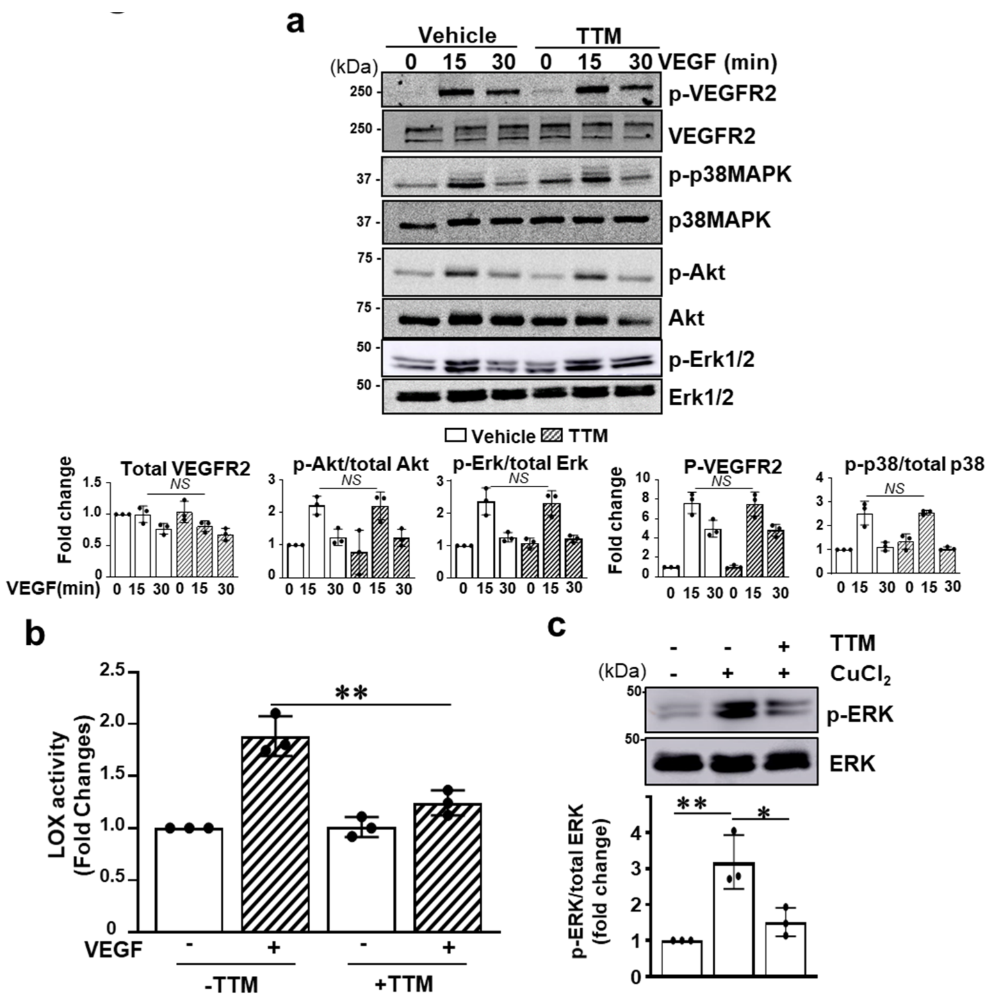

Supplementary Fig. 4: Cu chelator TTM has no effects on VEGFR2 signaling while it inhibits VEGF-induced LOX activity and Cu-induced p-ERK in ECs: a Human umbilical vein endothelial cells (HUVECs) pretreated with tetrathiomolybdate (TTM) (10nM) for 24hr were stimulated with vascular endothelial growth factor (VEGF) for indicated time. Lysates were immunoblotted (IB) with indicated Abs. n=3, NS=non-significant (two-tailed unpaired t-test). **b.** HUVECs pretreated with TTM (10nM) for 24hr were stimulated with VEGF (20ng/ml), and LOX activity was measured. n=3, **p=0.008 (two-tailed unpaired t-test). **c.** HUVECs pretreated with TTM (10nM) for 24h were stimulated with $CuCl_2$ (50uM) for 30 min and lysates were IB with indicated Abs. n=3, *p=0.0272, **p=0.0073 (two-tailed unpaired t-test). Data are mean ± SEM.

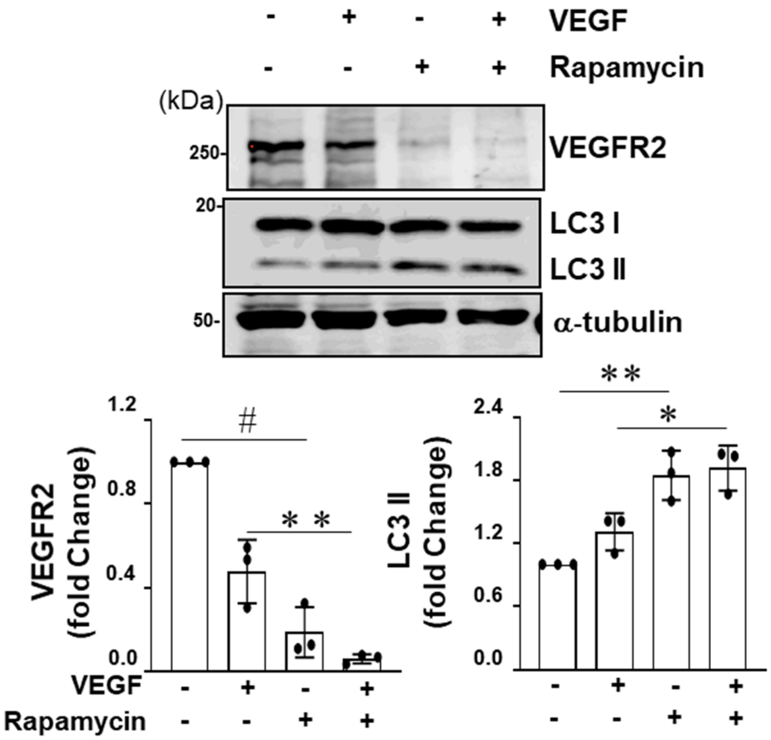

Supplementary Fig. 5: Rapamycin induced autophagy promotes VEGFR2 degradation: Human umbilical vein endothelial cells (HUVECs) were pretreated with rapamycin (10µM) for 12hr, followed by vascular endothelial growth factor (VEGF) (20ng/ml) stimulation for 60 min. Lysates were immunoblotted with anti-VEGFR2, LC3, or tubulin antibodies. n=3, VEGFR2: **p=0.0093, #p=0.0003; LC3II: *p=0.0195, **p=0.0033 (two-tailed unpaired t-test). Data are mean ± SEM.

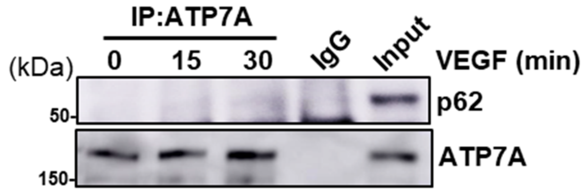

Supplementary Fig. 6: ATP7A does not bind to p62 in ECs stimulated with VEGF: Human umbilical vein endothelial cells (HUVECs) stimulated with 20 ng/ml vascular endothelial growth factor (VEGF) for indicated time were immunoprecipitated (IP) with anti-ATP7A antibody (Ab) or IgG (negative control), followed by immunoblotting with anti-p62 or ATP7A Abs. Input indicates lysates without IP. Blots are representative for three independent experiments.

Supplementary Table 1

Target gene	Primer Sequence
Human KDR (VEGFR2)	F - 5'-GGAACCTCACTATCCGCAGAGT-3'<br>R - 5'-CCAAGTTCGTCTTTTCCTGGGC-3'
Mouse ATP7A	Primers purchased from Qiagen with Cat # Mm_Atp7a_1_SG QuantiTect Primer Assay (QT00152677) (sequence not available)
